# Vocal cord movement: can it be accurately graded?

**DOI:** 10.1308/rcsann.2022.0091

**Published:** 2022-10-20

**Authors:** CM Douglas, R Menon, J Montgomery, R Townsley, O Hilmi, MA Buchanan, S Robertson, L Petropoulakis, JJ Soraghan, H Lakany, K Mackenzie

**Affiliations:** ^1^ NHS Greater Glasgow and Clyde, UK; ^2^ University of Strathclyde, UK; ^3^ NHS Ayrshire & Arran, UK

**Keywords:** Vocal cord movement, Vocal cord paralysis, Subjective assessment

## Abstract

**Introduction:**

Flexible nasendoscopy (FNE) is the principal assessment method for vocal cord movement. Because the procedure is inherently subjective it may not be possible for clinicians to grade the degree of vocal cord movement reliably. The aim of this study was to assess the accuracy and consistency of grading vocal cord movement as viewed via FNE.

**Methods:**

Thirty FNE videos, without sound or clinical information, were assessed by six consultant head and neck surgeons. The surgeons were asked to assess and grade right and left vocal cord movement independently, based on a five-category scale. This process was repeated three times on separate occasions. Agreement and reliability were assessed.

**Results:**

Mean overall observed inter-rater agreement was 67.7% (sd 1.9) with the five-category scale, increasing to 91.4% (sd 1.9) when a three-category scale was derived. Mean overall observed intra-rater agreement was 78.3% (sd 9.7) for five categories, increasing to 93.1% (sd 3.3) for three categories. Discriminating vocal cord motion was less reliable using the five-category scale (*k *= 0.52) than with the three-category scale (*k *= 0.68).

**Conclusions:**

This study demonstrates quantitatively that it is challenging to accurately and consistently grade subtle differences in vocal cord movement, as proven by the reduced agreement and reliability when using a five-point scale instead of a three-point scale. The study highlights the need for an objective measure to help in the assessment of vocal cord movement.

## Introduction

Flexible nasendoscopy (FNE) is the most commonly performed examination for the assessment of vocal cord movement. It is performed routinely on patients with voice complaints, and patient management is frequently based on the findings. FNE is the current gold standard for the evaluation of vocal cord motion, helping to distinguish between normal and reduced vocal cord movement. However, this subjective assessment can lead to inaccuracies and variability in diagnosis, especially in challenging cases. There is no reliable objective measure of categorising vocal cord movement from normal to complete paralysis. Furthermore, limited data exist on how consistent otolaryngologists are at rating vocal cord movement.^1,2^

The reliability of clinicians differentiating between binary categories of mobile and immobile vocal cords^1,2^ and the presence or absence of paresis have been reported.^3^ Madden and Rosen^1^ and Roscow and Sulica^2^ reported 95% inter-rater reliability and 99% intra-rater agreement for binary vocal cord assessment, and Estes *et al*^3^ an inter-rater reliability of 0.334 (Fleiss’s kappa). A three-category scale (paralysis, paresis, normal) has been used in reliability studies in paediatric patients.^4^ Liu *et al*^4^ reported an inter-rater reliability (Cohen’s kappa) of 0.67 for the diagnosis of normal versus impaired movement, and a lower reliability of 0.49 when identifying the degree of movement (normal, paresis, paralysis). Intra-rater reliability ranged from 0.48 to 1 (Fleiss’s kappa). There is currently no reliable grading system for categorising vocal cord movement from normal to complete paralysis; e.g. similar to the House Brackman scale used to routinely grade facial nerve paralysis.

The aim of this study was to determine whether experienced consultant head and neck surgeons were accurate and consistent in their assessment and grading of vocal cord movement.

## Methods

Thirty fibreoptic FNE videos of laryngeal movement were captured in a laryngology clinic. Cases ranged from normal vocal cord movement to complete laryngeal paralysis (nine normal; four palsies; three nodules; two each of cysts, functional dysphonia and inflammation; one each of Reinke’s oedema, presbyphonia, polyp, hypopharynx lesion, supraglottic lesion, crescentic defect of vocal cord, weakness and slower right vocal cord movement). The videos were pre-processed to reduce the effect of the honeycomb artefact caused by the fibreoptic endoscopes.^5^ Six consultant head and neck surgeons (JM, RT, OH, MB, SR, KM) were asked to subjectively assess vocal cord motion by visual inspection of the laryngeal videos and to individually rate the movement of the left and right vocal cords independently on a scale of 0 to 4 (Table 1). No clinical history or sound was associated with the videos. This process was repeated with the same videos, in a different order, on three separate occasions with a minimum of two weeks between each rating session. Each consultant rated the videos three times giving a total of 180 individual ratings (2 [right and left cord] × 30 × 3 = 180) per consultant and 1,080 (180 × 6 consultants) ratings in total. The consultants were blinded to their previous scores and those of other raters. Ethical approval was not required for this study.

**Table 1:** Rating scale used by the consultants

Score	Definition
0	No motion: The vocal cord is completely paralysed and shows no movement at all.
1	Almost no motion: The vocal cord is not completely paralysed, but shows only very slight movement.
2	Half the range of motion: The vocal cord moves about half the range of motion of that of a healthy vocal cord.
3	Almost full motion: The vocal cord moves with almost full range of motion, but not completely.
4	Full range of motion: The vocal cord moves completely with full range of motion.

### Statistical analysis

#### Agreement

Agreement was computed using the ‘percentage agreement’ measure, which provides the percentage of cases in which two or more raters scored identically. To assess inter-rater agreement, two percentage agreement measures were computed: the overall agreement between raters for all categories combined (overall percentage agreement); and the agreement specific to a category (specific agreement). The purpose of ‘specific agreement’ is to objectively demonstrate whether clinicians are in better agreement when rating cases belonging to some categories than others (such as the fully mobile category as opposed to paresis). Intra-rater agreement (test–retest) was also computed for each consultant over the three sessions using overall percentage agreement.

#### Reliability

Inter- and intra-rater reliability were calculated using the generalised Fleiss’s kappa^4,6,7^ to compare with equivalent studies reported in the literature. The kappa statistic ranges from 0 to 1, where 0 depicts that raters are in agreement only by chance. Any value over 0 may be interpreted as representing: poor (<0.40), fair to good (0.40–0.75) and excellent (>0.75) agreement beyond chance. The rating scale was considered as an ordinal scale and an ordinal weighting scheme was used in the computation of Fleiss’s kappa.^4,7^

For the intra-rater study, there were three sessions (replicates) per sample, which is appropriate^8,9^ because moderately high (>0.60) reliability was expected based on the trend in the literature.^1,3,4^ Because reliability was expected to be lower in the inter-rater study (as low as 0.33^3^), six raters are appropriate.^10^

### Rating scales

The study was conducted using a five-category scale (Table 1) to determine whether subtle differences in vocal cord motion can be visualised consistently between clinicians. It goes beyond the routine practice of describing motion as normal, paresis and paralysis, which is effectively a three-category scale. Hence a three-category scale was derived from the original five-category scale to determine the agreement/reliability using categories (normal/paresis/paralysis) that clinicians would normally use. This allows comparison between the three- and five-category scales. The re-categorisation from five to three categories was as follows: scores assigned to categories 0 and 1 were grouped together to form the ‘immobile’ category; scores assigned to categories 3 and 4 were grouped together to form the ‘fully mobile’ category; and category 2 remained effectively a ‘paresis’ category, resulting in the derived clinically relevant three-category scale. Inter- and intra-rater agreement and reliability measures were repeated using the derived scale.

### Ethical considerations

United Kingdom research ethics committee advice was sought using the online tool from the NHS health research authority and Medical Research Council website and was not required.^11^

## Results

All six consultants completed all the video assessments, giving a total of 1,080 individual vocal cord assessments. The results for the recorded five-category and derived three-category scales are reported.

### Agreement measures

The exact agreement in scores between the consultants, averaged over the three sessions is provided in Table 2. The overall percentage of observed inter-rater agreement, as shown in Table 2, was consistent across sessions with a mean value of 67.7% with the five-category scale, increasing to 91.4% when the three-category scale was used.

**Table 2:** Overall per cent agreement

Rating scale↓	Inter-rater agreement (%)		Intra-rater agreement (%)	
	Mean	sd	Mean	sd
Five categories	67.7	1.9	78.3	9.7
Three categories	91.4	1.9	93.1	3.3

Mean inter-rater agreement is the agreement between consultants in a given session, averaged over the three sessions. Mean intra-rater agreement is the agreement in the scores of a consultant between the three sessions, averaged over all consultants

sd = standard deviation above or below the mean

There was greater variability in the performance of the consultants in the five-category intra-rater study, with overall percentage agreement for a consultant between the three sessions ranging from 63.9% to 88.9%. Mean intra-rater agreement for the six consultants was 78.3% (sd 9.7%). With the three-category case, not only did the mean intra-rater agreement improve by 14.8% to give 93.1% agreement, but the variability in performance between consultants reduced, as shown by the threefold reduction in the standard deviation of the mean agreement measure.

The specific agreement between consultants for each category, averaged over the three sessions, is provided in Table 3.

**Table 3:** Inter-rater specific agreement (%)

Rating scale↓	Inter-rater agreement (±sd)				
Five categories	Immobile	Minimum residual mobility	Paresis	Slightly reduced mobility	Fully mobile
	58.6 (8.4)	16.7 (10.1)	23.9 (5.9)	22.8 (7.1)	83.1 (1.5)
Three categories	Immobile		Paresis	Fully mobile	
	75.1 (4.3)		23.9 (5.9)	96.1 (0.9)	

### Reliability measures

The consistency of discriminating vocal cord motion between the consultants (inter-rater) and between sessions for a given consultant (intra-rater) is provided in Table 4. Kappa values were consistent across sessions and the reported inter-rater reliability is the mean reliability of all sessions. Discriminating vocal cord motion was less reliable using the five-category scale (*κ *= 0.52) than with the three-category scale (*κ *= 0.68).

**Table 4:** Reliability measures

Rating scale↓	Inter-rater reliability: Fleiss’s kappa (±sd)	Intra-rater reliability: Fleiss’s kappa (±sd)
Five categories	0.52 (0.03)	0.69 (0.11)
Three categories	0.68 (0.06)	0.75 (0.1)

The intra-rater or test–retest reliability is the mean reliability of each consultant over the three sessions. With the five-category scale, intra-rater reliability ranged from 0.55 (fair) to 0.82 (excellent), with a mean of 0.69. Kappa values increased with the three-category scale and ranged from 0.64 to 0.87, with a mean of 0.75. Two of the six consultants had excellent reliability (0.78 and 0.82) with the five-category scale and three consultants had excellent reliability with the three-category scale (0.78, 0.87 and 0.87).

## Discussion

Correct diagnosis of a vocal cord movement abnormality is vital to help guide management of the patient, with potential medicolegal implications if misdiagnosed. There are many causes of abnormal movement, with movement ranging from fully mobile, to paresis to complete paralysis. Ideally, clinical assessment would result in a reliable five-category scale to allow use in a range of clinical situations such as reduction in movement in early invasive cancer or post thyroid surgery. Although the current gold standard for assessing the movement of a vocal cord is FNE, there are few published studies assessing consistency between different raters.

### Comparison between raters

#### Agreement measures

The six raters were asked to assess movement on a five-point scale, ranging from no movement to fully mobile. Inter-rater specific agreement was <60% for four of the five categories; immobile, slightly reduced movement, minimal residual mobility and paresis. The only category to have a high inter-rater specific agreement of 83.04%, was the fully mobile category. This may simply be because this is what clinicians see most commonly when performing FNE – a fully mobile vocal cord – with the high agreement being a reflection of pattern recognition. Furthermore, because the data set was formed from routine clinical cases, about 70% are of fully mobile vocal cords. Therefore, owing to the high prevalence, the positive predictive value of the clinicians for this score category would be high.^12^ Furthermore, when assessing each individual rating in the five-point scale, the combined agreement measure in each category varied considerably, from only 16.6% for score 1 (minimal movement) to 83% for score 4 (fully mobile). This significant range in agreement highlights the difficulty in assessing vocal cord mobility. When the options are limited to three categories, there was improved inter-rater specific agreement, with fully mobile agreement at 96.11%, and no mobility at 75.11%.

Analysis of specific agreement scores provides an insight into the categories for which the consultants were in greater agreement and the reason for the improvement in scores with the three-category scale. Clearly, much of the variability in scoring between clinicians is in categories 1 (minimal residual mobility), 2 (paresis) and 3 (slightly reduced mobility) in the five-category scale. The agreement in these categories for any session was <31%.

#### Reliability measures

Consistency in discriminating vocal cord motion between consultants was assessed. Discriminating vocal cord motion was less reliable using the five-category scale (*κ *= 0.52) compared with using the three-category scale (*κ *= 0.68), with both values falling in the fair to good grouping of reliability measures.^12^ Liu *et al*, when assessing paediatric patients, reported a reliability of *k* = 0.49 for three categories.^4^ Assuming that nasendoscopy is more challenging in the paediatric population and that Liu *et al* also did not use audio, our results seem comparable. Madden and Rosen reported higher inter-rater reliability of 95%, but they used a binary scale, i.e. purposeful vocal fold motion or no purposeful vocal fold motion, and their video data included audio.^1^ Nevertheless, Rosow and Sulica, who also included audio and employed a binary scale, reported the reliability of identifying the presence or absence of volitional adduction as only *k *= 0.335.^2^ However, their assessment was based on stroboscopy making it difficult to draw any firm comparisons.

### Repeatability of assessment

Consistency of re-examination affects clinical outcome and management decisions. When the five-point scale is used, it is clear that the intra-rater consistency is lower compared with the three-point score.

The diagnosis of vocal cord paresis is felt to be more challenging than vocal cord paralysis.^13^ This is highlighted in this study with low inter-rater specific agreement for scores 1, 2 and 3 in the five-point scale, and score 1 in the three-point scale (Table 3), which demonstrates that clinicians disagree with what they are seeing when vocal cord paresis is present. Vocal cord movement is a continuum with paresis not as well recognised or studied as paralysis. Wu and Sulica highlighted that in laryngology practice in North America, the most common diagnostic tool for diagnosing paresis was stroboscopy, not FNE.^13^ Simpson *et al*^14^ reported that in a large series of 739 patients presenting to a tertiary laryngology service with a chief complaint of dysphonia, of the 26.4% with paresis or paralysis on stroboscopy, only 1.8% of the patients had laryngeal electromyography (LEMG) confirmed vocal fold paresis. In stark comparison, Sataloff *et al*^15^ demonstrated that in his series of 689 patients with suspected paresis or paralysis, that LEMG confirmed this diagnosis in 95.9% of the patients. This significant variation between diagnosis and confirmation on LEMG highlights that we are still not able to consistently differentiate between these diagnoses. Although LEMG is the only way to confirm definitively that a patient has a paralysis or paresis, it is not routinely performed in clinical practice.

### Limitations of the study

This study aimed at assessing the consistency of clinicians evaluating movement of the vocal cord on a rating scale. Ideally, clinical assessment of voice should be multimodal and include voice recording, stroboscopic video analysis and electromyography recordings. These assessments should be used along with optical assessment in the form of FNE to ensure a full vocal assessment. ‘Worst-case scenario’ clinical situations were used, in which the clinician had no history from the patient and was unable to hear the patient’s voice when they assessed the video of vocal cord movement. Although not hearing voice quality is a limitation in the methodology of this study, this was necessary because it was the isolated subjective task of grading of vocal cord mobility without the distraction of hearing the effects of co-existent pathology that needed assessment. Because this is not representative of full clinical assessment, multimodal assessment of voice, taking account of the results of this study, should be considered in future studies. There was no extra information asked on the numerous other clinical findings that are seen in patients with vocal fold paralysis such as arytenoid prolapse, posterior gap, height and length mismatch.

The wide variation in inter-rater scores for the five-point scale may be related to the fact that there was no accompanying clinical history or sound with the videos, making it an artificial situation. Madden and Rosen,^1^ when assessing consistency of vocal fold motion, included sound with their videos and demonstrated higher inter-rater reliability, suggesting that a ‘complete picture’ is required when assessing vocal cord movement. All the endoscopies performed were fibreoptic FNE, which rendered poorer video quality than newer generation distal chip views, possibly making the more subtle movement of the vocal cords more difficult to judge and categorise. However, the videos very much reflected the reality of seeing patients in clinics and wards.

## Conclusion

This study demonstrates quantitatively that it is challenging to accurately and consistently grade subtle differences in vocal cord movement, as proven by the reduced agreement and reliability when using a five-point scale instead of a three-point scale. Therefore, this study highlights the need to have an objective measure to improve the accuracy of assessment of vocal cord movement. Image processing of endoscopy videos could be employed for measurement of vocal cord movement symmetry to quantify the degree of vocal cord motion, thus providing a reliable measure to assist in diagnosis and evaluate post-treatment outcomes.

## Conflicts of interest

The authors declare no competing interests.
